# Systems biology approaches to investigate the role of granulomas in TB-HIV coinfection

**DOI:** 10.3389/fimmu.2022.1014515

**Published:** 2022-10-31

**Authors:** Alexis Hoerter, Eusondia Arnett, Larry S. Schlesinger, Elsje Pienaar

**Affiliations:** ^1^ Weldon School of Biomedical Engineering, Purdue University, West Lafayette, IN, United States; ^2^ Host-Pathogen Interactions Program, Texas Biomedical Research Institute, San Antonio, TX, United States; ^3^ Regenstrief Center for Healthcare Engineering, Purdue University, West Lafayette, IN, United States

**Keywords:** tuberculosis, TB-HIV coinfection, granuloma, systems biology, HIV - human immunodeficiency virus

## Abstract

The risk of active tuberculosis disease is 15-21 times higher in those coinfected with human immunodeficiency virus-1 (HIV) compared to tuberculosis alone, and tuberculosis is the leading cause of death in HIV+ individuals. Mechanisms driving synergy between *Mycobacterium tuberculosis* (*Mtb*) and HIV during coinfection include: disruption of cytokine balances, impairment of innate and adaptive immune cell functionality, and *Mtb*-induced increase in HIV viral loads. Tuberculosis granulomas are the interface of host-pathogen interactions. Thus, granuloma-based research elucidating the role and relative impact of coinfection mechanisms within *Mtb* granulomas could inform cohesive treatments that target both pathogens simultaneously. We review known interactions between *Mtb* and HIV, and discuss how the structure, function and development of the granuloma microenvironment create a positive feedback loop favoring pathogen expansion and interaction. We also identify key outstanding questions and highlight how coupling computational modeling with *in vitro* and *in vivo* efforts could accelerate *Mtb*-HIV coinfection discoveries.

## Introduction

The synergistic interaction between *Mycobacterium tuberculosis* (*Mtb*) and human immunodeficiency virus-1 (HIV) has exacerbated the global burden of both diseases. A quarter of the global population is estimated to be infected with *Mtb* – the causative agent of tuberculosis (TB). TB deaths increased for the first time in a decade in 2020, due to COVID-related disruptions in care (1). There is a 5-10% lifetime risk of developing active TB disease following exposure to *Mtb*, and the risk of developing TB for HIV+ individuals is 15-21 times higher than for HIV– individuals (1). TB is the leading cause of death in HIV+ individuals claiming approximately 214,000 lives in 2020 (1). In addition to these pathological interactions between TB and HIV, there is also significant epidemiological overlap in these two infections. On the WHO’s lists of 30 high burden countries for TB and HIV-associated TB, 12 countries overlap (1). HIV coinfection also interferes with TB diagnostics including tuberculin skin tests and interferon-γ release assays (2). Thus, there is a need to focus on *Mtb*-HIV coinfection if we are to maintain hard-won gains in TB and HIV control.

While treatments exist for both TB and HIV, outcomes are not always positive in coinfected patients. Antiretroviral therapy (ART) in coinfected patients can reduce HIV-associated TB incidence (3). Nonetheless, alveolar macrophages, blood monocytes, and *Mtb*-specific T cells still have impaired functions or only partial restoration of function after ART initiation (4, 5). Paradoxical reactions following rapid immune reconstitution under ART, known as TB-immune reconstitution inflammatory syndrome (TB-IRIS), can also cause unintended clinical deterioration in coinfected patients [reviewed in (6)]. Drug-drug interactions as well as TB-IRIS can reduce efficacy of TB antibiotics and ART [reviewed in (7)]. To improve outcomes in coinfected patients we must develop treatments and vaccines specifically for *Mtb*-HIV coinfection, instead of each infection individually. Such treatments and vaccines will depend on a deeper understanding of the mechanisms of interaction between *Mtb* and HIV, and how these interactions combine to affect disease progression and treatment response.

*Mtb* infection is associated with a wide spectrum of outcomes ranging from complete elimination of bacteria, to bacterial containment in asymptomatic clinical states, to high bacterial replication in active disease with severe clinical symptoms (8). How individuals move throughout this spectrum is still being uncovered. It is believed that the clinical state of a patient is the combined result of the histopathologic response of multiple granulomas of different size and activity (9). Granulomas are the sites of infection of *Mtb* and consist of host immune cells, bacteria and dead cell debris. Granulomas exhibit a high level of heterogeneity between and within individuals, and can range from contained to disseminating (9, 10). Since granulomas are the primary site of infection of *Mtb*, we will focus our discussion on how the structure, timing and development of *Mtb* granulomas facilitates the synergism between *Mtb* and HIV.

*Mtb* granulomas develop in the lungs and lymph nodes following primary infection through a complex series of host-pathogen interactions. Granuloma formation is initiated through the recruitment of neutrophils, monocytes, macrophages and dendritic cells to the site of infection (11). This recruitment allows the host to mount an innate immune response, but also allows the bacteria to continuously infect macrophages, thus expanding their intracellular growth and dissemination (12). Following phagocytosis of *Mtb*, dendritic cells will migrate to the lymph nodes where they process and present antigen to *Mtb*-specific T cells. This process initiates the adaptive immune response where activated T cells are recruited to the site of *Mtb* infection through cytokine and chemokine production (13). The transition from the innate immune response to the adaptive immune response occurs about two to three weeks after infection by the ingress to the granuloma of lymphocytes (B and T cells) that form a lymphocyte cuff around the mass of macrophages (14). Germinal centers and B cell follicles have also been shown to contribute to granuloma structure formation (15). The arrival of lymphocytes can mark the end of rapid bacterial growth as the granuloma traps bacteria and can prevent its spread throughout the body. However, granulomas can also provide a replicative niche for the bacteria to persist inside and outside of the lungs (10). The organization of granulomas and long-term control of bacteria is dependent upon the recruitment of macrophages, CD4+ and CD8+ T cells, but also the cytokines that they secrete such as IFNγ and TNFα (16, 17). Caseous necrosis can form in the centers of granulomas as the result of the accumulation of dead cell debris. This necrotic region has been shown to be hypoxic (18) and hospitable to drug-tolerant and drug-resistant bacteria (19). It is within this complex and dynamic granuloma environment that we will discuss *Mtb*-HIV interactions.

Studying human granulomas is challenging. A systematic review and meta-analysis performed by Diedrich et al., 2016 (20) identified that there was a high degree of contrast among studies investigating the impact of HIV on *Mtb* granulomas during coinfection. Their analysis considered HIV impacts on granuloma formation, bacterial load, cellular composition, and cytokine production in *Mtb*-infected tissue from HIV+ and HIV– individuals. The authors found that the only consistent finding across studies was increased bacterial load in *Mtb*-HIV coinfected persons compared to *Mtb* infection alone (20). The authors could not conclusively confirm or reject any suggested hypotheses regarding *Mtb*-HIV coinfected granulomas. Two major sources of variation between studies are: a) granuloma descriptions (including location- lung vs lymph node) and scoring systems used, and b) the status of either disease stage, order of infections, and treatment status of the patients being studied. To address some of these challenges, non-human primate (NHP) models of coinfection use *Mtb* infection together with Simian immunodeficiency virus (SIV), which closely resembles human coinfection dynamics (21–32). NHP models offer the advantage of controlling the order of infection and knowing the status of the initial infection as well as treatment status.

A variety of mechanisms have been associated with the increased risk of developing active TB in HIV+ individuals. One major mechanism relates to the HIV-associated loss of macrophages and CD4+ T cells, both of which are instrumental in containing *Mtb* growth in granulomas (33). Since the hallmark of HIV infection is the slow depletion of CD4+ T cells over time, it has been suggested that CD4+ T cells in the granulomas of *Mtb* infected patients could provide a large source of target cells for HIV infection, and that their depletion directly interferes with granuloma control (34, 35). Foreman et al., 2022 (35) has demonstrated that CD4+ T cells within granulomas are directly depleted within 2 weeks following SIV coinfection of NHPs with LTBI before any detectable changes in CD4+ T cell counts in peripheral locations. This directly supports evidence that HIV-infected individuals have shown a 2- to 3- fold increase in the transition to active TB within the first year of HIV coinfection when CD4+ T cells are still relatively abundant (36). Therefore, HIV infection increases TB susceptibility, at least in part, independently of peripheral CD4+ T cell count, the hallmark of HIV progression (21, 37, 38). Furthermore, NHP models have demonstrated that CD4+ T cell depletion alone is not the sole mechanism responsible for the greater risk of active TB during HIV coinfection, since antibody mediated T cell depletion is not sufficient to drive LTBI to active TB, and not all NHPs with HIV-mediated T cell depletion develop active TB (21–23, 25, 26, 30). Therefore, it is necessary to evaluate additional mechanisms other than CD4+ T cell decline as possible causes for bacterial growth and dissemination, granuloma disruption, and progression of both infections in *Mtb*-HIV coinfected individuals.

Mechanisms in addition to CD4+ T cell depletion that have been implicated in the synergistic interactions between *Mtb* and HIV include: disruption of the pro- and anti-inflammatory cytokine balance, impairment of CD4+ T cell and *Mtb*-specific T cell functionality, *Mtb*-induced increase in HIV viral load, viral evolution within granulomas, chronic immune activation, and HIV impairment of innate immune cells (23–26, 28, 30, 32, 35, 39, 40). However, the role and relative impact of all of these mechanisms within *Mtb* granulomas remains unclear.

Discussion surrounding *Mtb*-HIV coinfection tends to focus on unidirectional impacts of one infection on the other. Instead we aim to focus on how each pathogen benefits from the other and from the microenvironment in which they both exist. Elucidating how *Mtb* and HIV are interfering with the immune response inside *Mtb* granulomas could inform new cohesive treatment strategies that target both pathogens simultaneously. The focus of this review is to show how the structure, function and development of the *Mtb* granuloma microenvironment favors bacterial and viral expansion during *Mtb*-HIV coinfection. Considerations of the impact of ART and TB treatments is important in *Mtb*-HIV coinfected individuals, but it adds a level of complexity outside the scope of this discussion (6, 7). Below, we present known interactions between *Mtb* and HIV and discuss how these might connect in the granuloma microenvironment to create a positive feedback loop beneficial towards disease progression. We identify key outstanding questions and propose that coupling computational modeling with ongoing *in vitro* and *in vivo* efforts will be key to understanding how these two pathogens are working synergistically to overcome the immune system.

## *Mtb*-HIV interactions in pre-existing TB infection

### It’s more than just a lack of CD4+ T cells

While CD4+ T cell depletion contributes to TB reactivation and progression (41), there are additional mechanisms, independent of CD4+ T cell depletion, that play a key role during *Mtb*-HIV coinfection (23, 25, 26). Diedrich et al., (21) was the first to use an NHP model (Chinese cynomolgus macaque) of *Mtb-*SIV coinfection. In this study, all LTBI NHPs experienced reactivation following SIV infection. Reactivation occurred across different time intervals and only when peripheral T cell numbers had returned to normal (21). The authors noted numerous completely fibrotic granulomas from NHPs that reactivated which is unusual in latent infection (21). Coinfected NHPs also had fewer CD4+ and CD8+ T cells in *Mtb*-involved lung tissue than SIV negative NHPs with active TB (21). Mattila et al., 2011 (22) extended the previous study to examine multifunctional T cell responses and granuloma T cell phenotypes in TB reactivated *Mtb*-SIV coinfected NHPs. *Mtb*-SIV coinfected NHPs experienced an increase in the frequency of *Mtb*-specific IFNγ and IL-2 expressing cells shortly after SIV infection and a sudden short-lived burst of IL-4 expressing cells (both CD4+ and CD8+) that was correlated with a period of reduced IFNγ and IL-2 expression. CD4+ T cell responses were not significantly different between early and late reactivating NHPs. However, early reactivating animals had significantly fewer Th0- (IFNγ^+^IL-4^+^IL-10^+^, IFNγ^+^IL-4^+^, IFNγ^+^IL-10^+^), fewer Th1- (IFNγ^+^) and more Th2-(IL-4+IL-10+, IL-4+, IL-10^+^) CD8+ T cells than late reactivating NHPs (22). In this study, they also observed that granuloma T cell responses were dominated by cytolytic T cell phenotypes compared to the cytokine-producing T cells being dominant in the periphery (22). Together, these studies highlight the involvement of CD8+ T cells, cytolytic T cells and inflammatory T cell phenotypes in *Mtb*-SIV coinfection progression. However, these studies don’t explicitly uncouple CD4+ T cell mediated impacts from SIV-mediated impacts.

Diedrich et al., 2020 (25) specifically determined the role of CD4+ T cell depletion by comparing *Mtb* infected NHPs who underwent antibody-mediated CD4 depletion (*Mtb*-αCD4), SIV coinfection, or no immune suppression (control). After 2 months, subclinical reactivation was observed in 5 of 7 *Mtb*-αCD4 NHPs and 4 of 8 *Mtb*-SIV coinfected NHP, suggesting similar rates of reactivation. *Mtb*-SIV coinfected NHP had more CD4+ T cells in granulomas compared to *Mtb*-αCD4. However, SIV-induced reactivation was associated with more disseminated lung granulomas and higher bacterial growth compared to *Mtb*-αCD4 NHPs that reactivated (25). Interestingly, the granulomas from *Mtb*-SIV coinfected NHPs had a more immune activated profile (more cytokines and granzyme B production) compared to *Mtb*-αCD4 NHPs. Granuloma composition was determined to be altered by *Mtb*-SIV coinfection and *Mtb*-αCD4 as noted by the presence of non-traditional CD3+ T cells found to be actively contributing to the overall immune function of granulomas in these groups. Further, the absolute number of CD8+ T cells within granulomas from *Mtb*-SIV NHPs was significantly greater than both LTBI control and *Mtb*-αCD4 NHPs. Together, these findings indicate that, despite having fewer CD4+ and CD8+ T cells as well as lower immune activation, *Mtb*-αCD4 NHPs were better able to control *Mtb* infections compared to *Mtb*-SIV coinfected NHPs. Sharan et al., 2022 showed that early events during SIV coinfection, prior to CD4+ T cell depletion, drives LTBI reactivation (42). This therefore implicates other SIV-associated impairments besides CD4+ T cell depletion, including cell type imbalances and inflammation, in facilitating TB reactivation and progression.

A similar study in Indian rhesus macaques compared *Mtb* infected NHPs with LTBI who underwent antibody-mediated CD4 depletion (CD4R1 administration), SIV coinfection, or no immune suppression (control) (26). Only 1 of 8 the CD4 depleted animals showed signs of reactivated TB while 9 of 16 *Mtb*-SIV coinfected NHPs reactivated. Despite this difference in reactivation rates, CD4-depleted and *Mtb*-SIV NHPs demonstrated similarly reduced CD4+ T cells in BAL (bronchoalveolar lavage) and lungs regardless of their TB outcome (26). Instead, coinfection with SIV resulted in a significant reduction of memory CD4+ T cells, which were replaced by naïve, not effector CD4+ T cells. Importantly, *Mtb*-SIV reactivators experienced higher levels of cytokines associated with chronic immune activation and inflammation (TNFα, IL1-α, IL-1β, and IL-6) and a more activated T cell phenotype (HLA-DR+) in the lungs compared with NHPs administered CD4R1 (26). These patterns were also observed in the CD8+ T cell compartment (26). The striking difference in reactivation rates between this Bucsan et al., 2019 (26) study and the earlier mentioned Diedrich et al., 2020 (25) study, could be attributed to the difference in NHP model used [cynomolgus (25) vs rhesus (26)) and different *Mtb* strains (Erdman (25) vs CDC1551 (26)], as noted by Diedrich et al., 2020 (25). Additionally, there were differences in the time frame to establish LTBI (LTBI established 9 weeks after *Mtb* inoculation (26) vs LTBI established 6 months after *Mtb* inoculation (25)) and the clinical endpoint for reactivation used (subclinical reactivation (appearance of new granuloma) defined by PET CT (25) vs overt clinical symptoms (26)). Nevertheless, this work supports the role of cell type and cell phenotype imbalances as well as inflammation and activation, not just CD4+ T cell depletion, in TB reactivation in *Mtb*-SIV coinfection.

Foreman et al., 2016 (23) explored the impact of SIV on LTBI by comparing *Mtb*-SIV coinfected NHPs termed reactivators (animals that progressed into active TB: 9/14) and non-reactivators (retained LTBI status 5/14) following SIV infection of LTBI NHPs. Reactivators had a) higher BAL *Mtb* CFU (colony-forming units) values compared to non-reactivators or LTBI only NHPs, b) similar culturable *Mtb* from the lungs compared to the active TB group, c) more disseminated *Mtb* than non-reactivators and LTBI only NHPs, and d) more pulmonary lesions than non-reactivators and LTBI only group. All *Mtb*-SIV coinfected NHPs showed signs of SIV-induced pulmonary pathology, but the extent of lung pathology was more severe in reactivators compared to non-reactivators (23). Similar to NHP studies previously mentioned in this review, there was massive reduction in CD4+ T cell percentages and absolute numbers. This reduction was observed in all coinfected NHPs (reactivators and non-reactivators) BAL samples. However, the reduction in CD4+ T cells in BAL was not significantly different between reactivators and non-reactivators, thereby implicating additional mechanisms beyond CD4+ T cell depletion in TB reactivation during coinfection. Qualitatively comparing subsets of the few remaining CD4+ T cells in BAL, a) there was no difference in central memory cells (percentage, absolute numbers, or turnover), b) reactivators had a significantly higher percentage of effector memory cells (and absolute number) but their turnover was not significant, and c) regulatory CD4+ T cells were significantly higher in non-reactivators (23). The authors noted that this increase in regulatory CD4+ T cells in non-reactivators concurrent with decreased effector memory CD4+ T cells suggests a role for these regulatory cells in limiting disease causing pathology (which was increased in reactivators). Other additional mechanisms beyond CD4+ T cell depletion identified include: higher rate of turnover (Ki67+) of CD8+ T cells in non-reactivators, indicating that these cells were active and proliferating; more well-organized areas of lymphoid follicles in non-reactivators; and, in contrast to Diedrich et al., 2020 (25), higher production of granzyme B in non-reactivators, indicating increased cytolytic activity.

There is also evidence that *Mtb*-specific CD4+ T cells play a role in increased disease in *Mtb*-HIV coinfection. It has been hypothesized that HIV preferentially targets *Mtb*-specific CD4+ T cells. Geldmacher et al., 2008 (37) has shown that fewer *Mtb-*specific CD4+ T cells existed in peripheral blood of LTBI individuals who became infected with HIV compared to HIV-uninfected patients with active TB (37). The same group also suggested that HIV preferentially targets *Mtb*-specific CD4+ T cells based on finding more HIV DNA in *Mtb*-specific CD4+ T cells compared to non-*Mtb*-specific CD4+ T cells in peripheral blood (43). The authors, as well as others, concluded that differences in the functions of *Mtb*-specific CD4+ T cells, particularly a weak ability to produce a natural antagonist to HIV CCR5 receptor entry, likely contributes to their increased susceptibility to HIV compared to *Mtb* non-specific cells, resulting in markedly depleted *Mtb*-specific CD4+ T cells (43). Other studies echo the decrease in frequency of *Mtb*-specific CD4+ T cells and highlight changes in functionality of *Mtb*-specific CD4+ T cells following *in vitro* studies of HIV+ individuals (44) as well as *in vivo* studies from LTBI-HIV infected individuals compared to HIV-uninfected individuals (45, 46). Day et al., 2017 found that blood samples from LTBI individuals with HIV infection had lower frequencies of cytokine-producing *Mtb*-specific CD4+ T cells, preferential depletion of a specific subset of polyfunctional *Mtb*-specific CD4+ T cells, and reduced proliferative capacity of *Mtb*-specific CD4+ T cells compared to HIV– individuals (45). Amelio et al., 2018 reported that Tanzanian individuals with *Mtb*-HIV coinfection had significantly fewer IL-4/IL-5 and IL-13 producing *Mtb-*specific CD4+ T cells and fewer IL-2 producing *Mtb-*specific CD4+ and CD8+ T cells compared to individuals with just TB. In addition to lower quantity, the authors showed that HIV suppresses *Mtb*-induced systemic proinflammatory cytokine responses (46). Another study in LTBI individuals with and without HIV found that *Mtb*-specific CD8+ T cell functionality was impaired in the HIV+ group as shown by decreased CD8+ T cell proliferation and degranulation activity (47). Thus, HIV appears to reduce the number of *Mtb*-specific T cells as well as the antimicrobial functionality of both CD4+ and CD8+ *Mtb*-specific T cells functionality. Foreman et al., 2022 (35), demonstrated in a rhesus macaque model that *Mtb*-specific CD4+ T cells are specifically decimated in granulomas from *Mtb*-SIV coinfected animals long before depletion is detectable in blood and that coinfection significantly decreased CD4+ T cell movement in granulomas (35).

In the context of *Mtb*-HIV coinfection, immune cell dynamics in the lungs are likely to have a large impact on *Mtb* infection progression (27, 30). Kuroda et al., 2018 (30) observed that monocyte and macrophage turnover rates in SIV-infected lung tissue were higher in animals that reactivated compared to those that remained LTBI (30). Corleis et al., 2019 (27) showed that during early SIV infection, loss of lung interstitial CD4+ T cells before loss of circulating CD4+ T cells is associated with increased dissemination of pulmonary *Mtb* infection. Early severe depletion of lung interstitial CD4+ T cells *in vitro*, induced by HIV in human cells was also determined in this study. HIV-induced lung interstitial CD4+ T cell depletion was accompanied by high virus production *in vitro* and the cells with the highest rate of virus production were the most severely depleted (27). Therefore, both numbers and turnover of innate and adaptive immune cells in lung tissue appear to be altered by *Mtb*-HIV coinfection compared to *Mtb* infection alone. These dynamics in the lungs are likely to impact granuloma formation and function.

Indeed, studies have demonstrated that the composition of granulomas from *Mtb*-HIV coinfected individuals is altered compared to *Mtb* infection alone. Spinal granulomas from coinfected individuals had more CD8+ T cells than TB only (48). Excised cervical lymph nodes of *Mtb*-HIV coinfected individuals had increased *Mtb* load and fewer CD4+ T cells compared to *Mtb* mono-infection (49). Unlike many of the NHP studies mentioned, peripheral CD4+ T-cell depletion correlated with granulomas that contained fewer CD4+ and CD8+ T cells, less IFNγ production, more neutrophils, more IL-10, and increased *Mtb* numbers (49). *Mtb* numbers correlated positively with IL-10 and IFNα levels and fewer CD4+ and CD8+ T cells (49). In a humanized mouse (HuMouse) model of *Mtb*-HIV coinfection and *Mtb*-HIV coinfected human lung tissues from autopsy, there was an accumulation of neutrophils localized to poorly organized inflammatory areas in coinfected tissues in human subjects and HuMice, as well as excessive pro-inflammatory cytokine responses in the pulmonary microenvironment compared to *Mtb*-only infected lungs (50).

Taken together, these studies illustrate that T cell numbers and function, along with cell localization in granulomas, macrophage turnover, and chronic/dysregulated inflammation all contribute to the deleterious effect of *Mtb-*HIV coinfection on TB progression. This highlights the notion that *Mtb*-HIV coinfection impacts TB progression in additional ways other than CD4+ T cells numbers both in granulomas and peripherally.

### TB granuloma microenvironment is permissive to HIV infection and replication

We next turn our attention to how the *Mtb* microenvironment might facilitate HIV infection progression and in turn *Mtb* replication. Many studies have shown that *Mtb* coinfection enhances HIV replication and infectivity *in vitro* and *in vivo (* 51–53). Larson et al., 2017 (54), even showed that *Mtb* coinfection can reactivate latent HIV infection (54). Higher levels of p24 (HIV viral antigen) were associated with BAL fluid from *Mtb*-involved (determined by chest radiographs) lung tissue compared to *Mtb*-uninvolved lung tissue from *Mtb-*HIV coinfected individuals (52, 55). HIV+ cells were localized almost exclusively to site of *Mtb* lesions compared to being occasionally found and distributed in the lung parenchyma during HIV only infection (50). Several studies have shown that pleural fluid from *Mtb*-HIV coinfected individuals with pleural TB had greater levels of HIV proteins compared to plasma fluid (56, 57). The number of HIV RNA-positive cells was 10-fold higher in lymphoid tissues with granulomas than without from *Mtb*-HIV coinfected individuals (58). In *Mtb*-SIV coinfected NHP models, Foreman et al., 2016 (23), showed that *Mtb*-SIV coinfected NHPs that had reactivated TB had higher numbers of SIV-infected cells in granulomas than *Mtb*-SIV coinfected non-reactivators (23). Kuroda et al., 2018 (30), observed high levels of SIV DNA in lung macrophages from reactivated *Mtb-*SIV coinfected NHPs but not in LTBI *Mtb-*SIV coinfected NHPs (30). Diedrich et al., 2020 (25), observed that *Mtb* CFU+ granulomas from *Mtb*-SIV coinfected NHPs were associated with higher SIV RNA compared to *Mtb* CFU- granulomas. Granulomas from *Mtb*-SIV coinfected NHP reactivators had higher SIV RNA than non-reactivators even when accounting for number of CD4+ T cells (25). Additionally, they also showed that granulomas with SIV RNA from *Mtb*-SIV coinfected animals contained higher *Mtb* counts than granulomas without SIV RNA (25). While the cause and effect relationships between increased bacterial growth/reactivation and higher HIV/SIV viral RNA is complex, these data show that a) *Mtb* sites of infection are associated with higher HIV viral loads compared to plasma viral loads in *Mtb*-HIV coinfected individuals and b) higher bacterial numbers or TB disease progression is associated with higher viral loads.

Recent advances indicate that *Mtb* infection increases viral replication via*Mtb*-induced inflammation (59–62). Along with greater levels of HIV proteins, greater concentrations of proinflammatory cytokines and immune activation markers were found in pleural fluid compared to plasma in coinfected patients with pleural TB (56). Rodriguez et al., 2013 (59) showed that PIM_6_, a major cell wall associated mycobacterial glycolipid, can increase HIV replication in CD4+ T cells independently of accessory cells (59). Similarly, Pouget et al., 2021 (60), observed that bacterial glycolipid antigens did not interfere with HIV entry directly, but that increased HIV replication is likely due to *Mtb* antigen-induced modulation of the immune system (60). However, the authors did show that *Mtb* glycolipids might potentially impair or enhance HIV trans-infection (DC capture and presentation of viral antigen to CD4+ T cells) of both R5 and X4 tropic HIV strains, which has important implications for virus transmission and dissemination (60). He et al., 2020 showed that *in vitro* infection with HIV of CD4+ T cells from individuals with LTBI were more efficient at HIV transcription (post-integration) than CD4+ T cells from active TB or *Mtb*-uninfected individuals (61). Monocyte chemoattractant protein-1 (MCP-1) has also been observed in high levels in pleural fluid from *Mtb*-HIV coinfected patients and is implicated in induction of HIV transcription in macrophages in *Mtb*-HIV coinfected individuals (62).

The cellular phenotypes present at the site of *Mtb* infection can also facilitate HIV replication (50, 63, 64). The chemokine receptors CXCR4 and CCR5 function as coreceptors for direct infection of CD4+ target cells by HIV (65). In *Mtb* infection, it has been reported that CCR5 expressing macrophages are predominant at the site of *Mtb* infection (66). CCR5 expressing T cells have also been implicated in instructing correct T cell localization in granulomas to promote macrophage activation thus suggesting their important role in protective outcomes in *Mtb* infection (67). Elevated expression of CXCR4 and CCR5 on macrophages/monocytes (66, 68) and CD4+ T cells (63), have been reported in patients with TB and after *in vitro* stimulation with *Mtb* antigen in cells from healthy subjects. Additionally, *Mtb* infection has been demonstrated to increase CXCR4 surface expression allowing a more permissive environment for X4 HIV strains (64). In terms of coinfection, a study comparing blood and pericardial fluid from TB patients with and without HIV found that a majority of CD4+ T cells from pericardial fluid of HIV-uninfected patients expressed CCR5 and were of effector memory and terminally differentiated phenotype while the *Mtb-*specific CD4+ T cells in the pericardial fluid from the coinfected group lacked the CCR5 receptor and were a less differentiated phenotype (69), suggesting that CCR5 positive cells are preferentially depleted. In contrast to this, in an LTBI NHP model comparing CD4+ T cell depletion to coinfection with SIV, Bucsan et al., 2019 (26) observed higher CCR5 T cells in the lungs of coinfected LTBI-SIV reactivator NHPs than just CD4 depleted-TB infected animals indicating higher recruitment to the primary site of infection in coinfected animals (26). The discrepancy between these two studies in terms of the impact of coinfection on CCR5 positive cells, could be due to the time frame of the infections: CCR5 cells might increase in the period after coinfection but might be preferentially depleted as time goes on. Foreman et al., 2022 (35), demonstrated that SIV coinfected NHP granulomas had reduced frequency of CCR5-expressing Th1 and Th1* cells 2 weeks after SIV coinfection (35). Taken together, the cellular phenotype present in the *Mtb* granuloma likely provides an abundance of cells targeted by HIV in various stages of HIV progression.

Increased HIV replication induced by *Mtb* infection may also be associated with an increased number of viral variants in *Mtb*-HIV coinfected patients compared to just HIV+ individuals (55, 70, 71). Collins et al., 2000 (70) showed that blood samples from *Mtb*-HIV coinfected individuals had increased HIV heterogeneity and significantly more mutations than HIV only infection (70). In a follow-up study, authors compared blood and pleural fluid from *Mtb*-HIV infected individuals investigating whether a TB-associated, site-specific increase in HIV replication and heterogeneity in the lung may migrate to the blood to increase systemic HIV heterogeneity. Their results indicated there is an increase in HIV genetic diversity in the pleural space compared to the blood and there is a unidirectional migration of quasispecies from the pleural space to the blood (71). Tisthammer et al., 2022 (29), was the first to assess *in vivo* SIV diversity in *Mtb*-SIV coinfected NHPs compared to SIV only in samples from plasma, lymph nodes, and lungs (including granulomas). Low viral diversity and the small number of animals with a limited number of samples for each tissue type made comparisons among groups difficult, but results showed that the highest viral diversity was among *Mtb*-SIV coinfected NHPs that did not develop clinical *Mtb* reactivation compared to those that did and that viral diversity was positively correlated with the frequency of CD4+ T cells and negatively correlated with the frequency of CD8+ T cells in lung granulomas of *Mtb*-SIV coinfected NHPs that developed clinical *Mtb* reactivation (29).

Collectively this paints a picture of *Mtb* being associated with increased viral loads through its impact on inflammation, recruitment/cellular phenotype and viral diversity.

## *Mtb-*HIV interactions in pre-existing HIV infection

Pre-existing HIV might have a different set of mechanisms responsible for an increase in *Mtb* growth and dissemination following subsequent TB coinfection. HIV infection is known to impact innate immune cell function [reviewed in (72)]. While HIV predominately infects CD4+ T cells, it can also infect macrophages and dendritic cells (73). Upon infecting alveolar macrophages, HIV impedes receptor-mediated phagocytosis and cell apoptosis which limits the cell’s ability to kill intracellular bacteria (74). In dendritic cells, HIV can disrupt *Mtb* antigen processing and presentation which alters the initiation of the adaptive immune response (74). It has been demonstrated in macrophage cultures that HIV coinfection of macrophages enhances *Mtb* growth (75). HIV infection significantly reduced *Mtb*-mediated macrophage apoptosis in alveolar macrophages from HIV+ individuals. This reduction in apoptosis has been linked to a decline in *Mtb*-mediated TNFα release from HIV+ macrophages by HIV Nef protein, thus reducing macrophages’ ability to kill intracellular bacteria (76). HIV infection was also demonstrated to inhibit the macrophages’ ability to acidify and fuse *Mtb*-infected phagosomes with lysosomes, a key step in the anti-bacterial responses of macrophages (77). Thus, HIV impairs multiple aspects of innate immune responses to *Mtb* infection. These innate responses drive the initial immune response to a new *Mtb* infection, which in turn can determine long term disease trajectories (78).

Indeed, Guo et al., 2017 (32) showed in a Chinese rhesus macaque NHP model, that pre-existing SIV infection led to an increase in extrapulmonary TB, higher bacterial counts in lungs, lymph nodes and extrapulmonary organs, and lower levels of IFNγ and IL-22 (cytokines known to inhibit *Mtb* infection) compared to *Mtb* infection alone (32). Rodgers et al., 2018 (24), showed in a Mauritian cynomolgus macaque model that pre-existing SIV infection accelerated TB infection progression with a significant increase in the number of TB granulomas between 4- and 8-weeks post *Mtb* infection compared to SIV-naïve, *Mtb*-infected animals. Coinfected animals had higher bacterial loads and an increased trend towards extrapulmonary TB compared to *Mtb*-infected animals (24). Larson et al., 2021 (28) extended the previous model to characterize the CD4+ and CD8+ T cell phenotypes and cytokine production in blood, airway and tissues of *Mtb*-SIV coinfected macaques compared to *Mtb* infection alone. They demonstrated that animals with pre-existing SIV infection had 1) lower CD4/CD8 T cell ratios across all tissue compartments including granulomas, 2) higher frequencies of CD4+ and CD8+ T cells expressing PD-1 or TIGIT (markers associated with immune activation) at sites of *Mtb* infection, and 3) fewer CD4+ T cells producing TNF in granulomas (28). Most recently, Moriarty et al., 2022 furthered the analysis of the previous two studies (Rodgers et al., 2018 and Larson et al., 2021) by dividing the coinfected NHPs into two groups, viral controllers and viral non-controllers. New conclusions from this analysis determined that pre-existing SIV infection, regardless of viral control, in *Mtb*-SIV coinfected NHPs results in enhanced *Mtb* dissemination and dysregulated T cell immune responses (40). SIV+ viral controllers had a significantly lower frequency of CD4+ T cells that were producing IFNγ. There was also a highly significant correlation between *Mtb* CFU and SIV copies/cell in *Mtb*-affected tissues across all SIV+ NHPs.

Taken together, these studies demonstrate that upon *Mtb* infection in animals with pre-existing SIV infection, coinfected animals have more *Mtb* dissemination, and higher bacterial loads compared to *Mtb* infection alone. These pathological differences arise from disruptions in both innate and adaptive immune responses to *Mtb*, including macrophage antimicrobial function, DC antigen processing and presentation, ratios of CD4/CD8 cells, and immune activation on both CD4+ and CD8+ T cells.

## Synergistic *Mtb*-HIV interactions in granulomas and outstanding questions

As a whole, the previous sections demonstrate that many complex interactions are involved in *Mtb*-HIV coinfection, and that coinfection facilitates and accelerates progression of both infections. Here we discuss the potential *collective* impact of these interactions within the context of TB granulomas.

Granulomas create a milieu of inflammation that enhances HIV replication within cells. The granuloma also constitutes a mixture of cells that are susceptible and permissive to HIV infection and replication - namely CCR5 and CXCR4 expressing cells - thereby facilitating viral spread between cells. This localized viral expansion then depletes CD4+ and *Mtb*-specific T cells within the granuloma, thereby disrupting the crucial balance between pro-and anti-inflammatory cytokine signals which is needed to establish and maintain control of bacterial growth (9, 17). NHP studies have suggested that the initial extent of T cell depletion might cause a significant disruption in the T cell population within granulomas that is unable to be reversed when peripheral T cells recover inflicting long-lasting consequences in the granuloma (21). The increase in bacterial growth resulting from this local T cell depletion will in turn increase inflammation and further immune cell recruitment to the granuloma. Since HIV infection seems to impair the number and functionality of *Mtb*-specific CD4+ T cells – both in the granuloma and peripherally – the newly recruited *Mtb*-specific CD4+ T cells might be ill-equipped to support anti-mycobacterial functions in the granulomas. This cellular recruitment could support viral expansion in the granuloma in two ways: a) by recruiting HIV+ cells and seeding new infections within the granuloma, and b) recruiting new target cells to support the continued viral replication within the granuloma. In this sense the granuloma is providing a permissive environment with a greater density and diversity of cells that support HIV expansion and fails to control *Mtb* replication.

**Figure 1:**
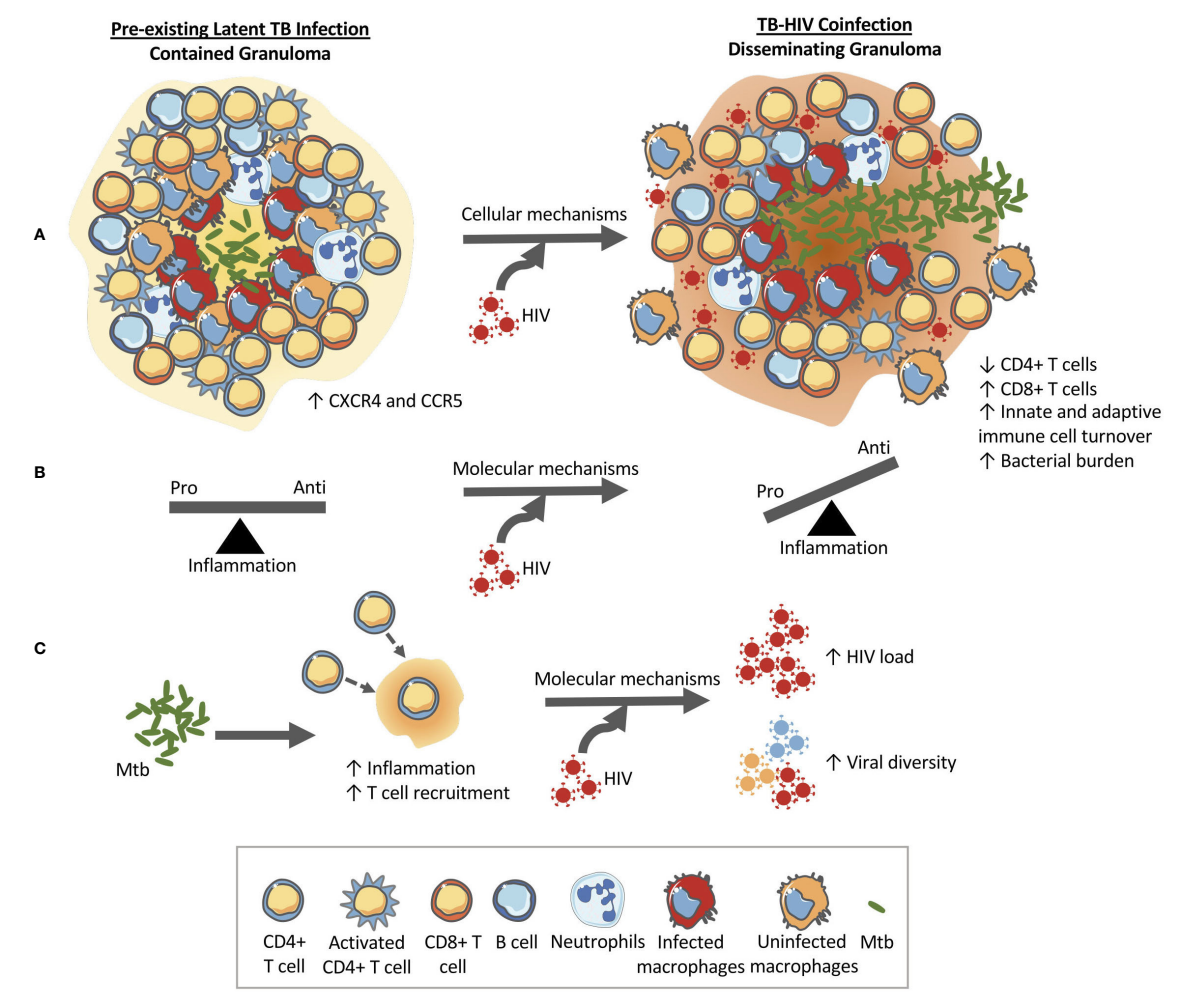
HIV changes the structure and function of the granuloma in pre-existing LTBI allowing for bacterial growth and dissemination. **(A)** Cellular level changes: An example granuloma classified as contained (bacteria localized) made up of CD4+ and CD8+ T cells, B cells, macrophages, and neutrophils that transforms into a disseminating granuloma upon HIV infection. The granuloma is rich in HIV susceptible cells (CXCR4 and CCR5 cells) that can help accelerate CD4+ T cell destruction. Granulomas during coinfection have been shown to contain higher CD8+ T cells and have higher rates of macrophage turnover. These cellular changes at the granuloma level ultimately alter the function of the granuloma which can lead to increased bacterial load and dissemination. **(B)** Molecular mechanism #1: HIV has been shown to have detrimental impacts on the functionality of CD4+ T cells and therefore the balance between pro-and anti-inflammatory cytokines produced by the local immune cells is thrown into dysregulation. The change in the cytokine milieu can facilitate granuloma dissolution. **(C)** Molecular mechanism #2: The immune response to *Mtb* results in an increase in inflammation and T cell recruitment to the site of infection. This *Mtb*-induced inflammation and availability of HIV susceptible cells within the granuloma (CXCR4 and CCR5 cells) have been implicated in the increased viral loads and increased viral diversity found in coinfection. An increase in viral load would lead to local destruction of the cells in the granuloma therefore aiding bacterial dissemination.

Increased macrophage turnover in lung tissue and in granulomas could further impact granuloma functionality by disrupting the spatial organization of typical granulomas where a macrophage core is surrounded by a cuff of macrophages mixed with T cells. This disorganization combined with lower frequencies of *Mtb*-specific T cells could also hamper the ability of *Mtb*-specific T cells to find and activate *Mtb*-infected macrophages due to disrupted chemokine gradients and obstruction by non-specific T cells (79). Thus, the combined impacts of *Mtb* and HIV within granulomas enable a positive feedback loop that exacerbates both infections. **Figure 1** shows how a contained granuloma in pre-existing LTBI might turn into a disseminating granuloma upon subsequent HIV coinfection through a change in inflammatory profile, CD4+ T cell functionality, and cellular composition of granulomas.

**Figure 2:**
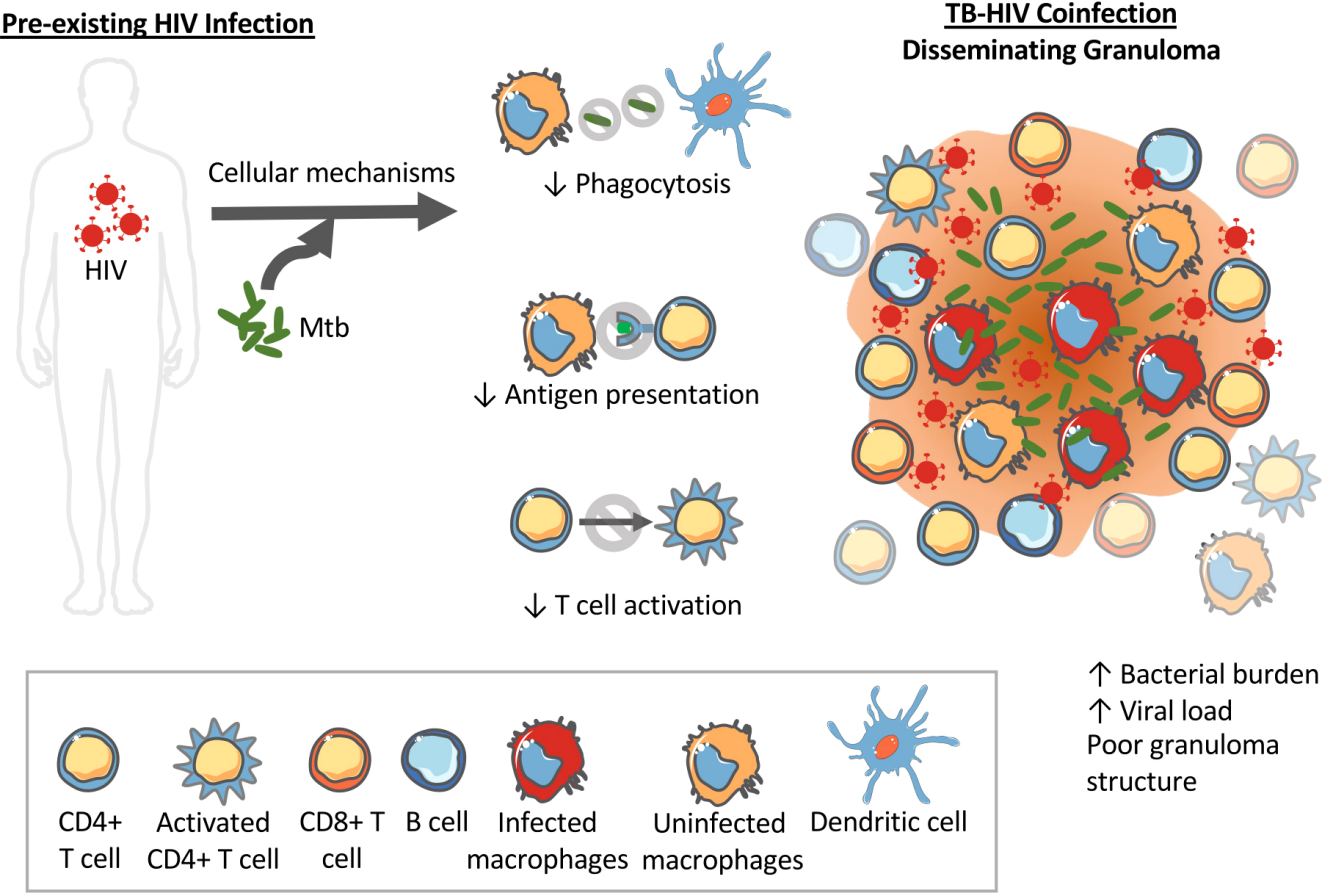
Pre-existing HIV impacts innate immune functions that can impact *Mtb* granuloma development and progression. HIV can alter the phagocytosis abilities of dendritic cells and macrophages limiting their ability to kill intracellular *Mtb*. HIV also interferes with antigen processing and presentation required to activate T cells. The result is disorganized granulomas with high bacterial load, high viral load, and high dissemination rates.

The impacts of pre-existing HIV on innate and adaptive immune cells could initiate a similar positive feedback loop disrupting granuloma formation, structure and bacterial control. For instance, upon exposure to *Mtb* in an HIV-infected individual, decreased phagocytosis by innate immune cells like macrophages and DCs would inhibit clearance of bacteria. It is also possible that HIV impacts on macrophages contribute to cytokine dysregulation. HIV-induced decreased antigen processing and presentation by DCs to T cells in the lymph nodes would inhibit and delay T cell activation. Indeed, NHP studies show a decrease in TNF-producing CD4+ T cells as early as 6 weeks post *Mtb* infection (28). Decreased T cell activation and recruitment to the lungs where bacteria have established would mean granulomas have fewer T cells and poorly contain bacteria. In addition to having poor phagocytosis abilities, the macrophages at the granulomas might be inefficient at killing bacteria due to HIV-induced macrophage dysfunction. Decreased phagocytosis, decreased bacterial killing and decreased T cells in the granulomas at the primary site of the *Mtb* infection would increase bacterial growth. This bacterial growth would again drive increased viral replication and spread. In this situation, the lack of a structured granuloma prevents the opportunity to establish latent TB infection and instead facilitates disseminated TB. The observation of increased CD8+ T cells in BAL and granulomas of coinfected NHPs could also be explained by *Mtb* induced activation of SIV-infected cells in the lungs and airways that in turn increases SIV replication and drives the influx of CD8+ T cells (28). **Figure 2** demonstrates how HIV coinfection drives bacterial growth and dissemination, and vice versa, in *Mtb*-HIV coinfected individuals with pre-existing HIV.

Taken together, the contextualization of *Mtb*-HIV interactions within granulomas (**Figures 1**, **2**) illustrate how granuloma dynamics could lead to the coinfection outcomes we observe at the tissue/host level. It’s important to note that the above discussions combine a few coinfection mechanisms to illustrate the positive feedback between *Mtb* and HIV infections. However, all of these mechanisms are most likely occurring simultaneously or with greater influence at different times or specific locations within the body. A number of questions regarding this complex network of interactions between *Mtb*, HIV and the host immune response and the role of granulomas in *Mtb-*HIV coinfection remain unanswered (**Box 1**). Answering these questions could move us toward interventions that prevent LTBI reactivation in patients that are newly coinfected with HIV or developing treatment strategies targeted to coinfected individuals.

As described in the literature discussions above, there is significant effort devoted to uncovering the numerous and complex ways in which *Mtb* and HIV interact. Nonetheless, a number of challenges remain. Currently it is a formidable task to study the very early dynamics of the granuloma *in vivo*. Conclusions are often based on end time points, which do not elucidate how a process happened – just that it happened. Different stages of disease can also impact what mechanism is the driving factor for disease progression. Investigating one mechanism at a specific time might incorrectly lead to the wrong conclusion because each interaction may have different consequences at different time points and in different stages of disease progression. Multiple tissue compartments (peripheral blood, lungs, lymph nodes, etc.), cells types (macrophages, DCs, CD4+ T cells, CD8+ T cells, etc.), and functions (phagocytosis, secretion, antigen processing/presentation, activation, apoptosis, etc.) are involved in this complex coinfection. It is not always feasible or possible to determine key metrics in each of these compartments or cell types. Investigating *Mtb*-HIV coinfection in human subjects has other challenges including difficulty in determining when and which infection came first and the status of the immune system pre-coinfection (21). There is limited availability of pre- and post-infection samples as unnecessary and invasive procedures are used to obtain samples (21). It is particularly difficult to study the sequence of events involved in the reactivation of LTBI since biomarkers of TB reactivation are still being elucidated (80, 81).

Thus, animal and *in vitro* models have been deemed a valuable asset to study coinfection dynamics as the timing of each infection can be controlled to explore early observations of immune responses to various immunological events (82). NHPs are the most suitable animal models that closely resemble human TB granulomas and disease states (developing active and LTBI) and coinfection dynamics but disadvantages include dedicated veterinarian staff required, cost, and ethical considerations (83, 84). For NHP models, similar to humans, there is also variability in what defines LTBI and what clinical or pathological markers are used to define disease progression. It has also been noted how different *Mtb* and HIV strains and doses, and species of macaques might lead to discrepancy among NHP studies since there were contrasting results among several NHP models (24, 32).

*In vitro* systems using human cells are valuable in analyzing host-pathogen interactions and disruptions but the necessary simplifications make it difficult to test multiple mechanisms at once and often have a limited number of cells from the same donor (85). Therefore, simultaneously quantifying and analyzing the impact of combinations of *Mtb*-HIV interactions creates an experimental design challenge. Computational models offer complementary attributes that can help alleviate these experimental challenges and bridge the gap between *in vivo* and *in vitro* experiments. For example, highly controlled studies that have singled out specific mechanisms can be combined into a complex and highly interconnected model.

**Box 1: Summary of outstanding questions related to the role of the granuloma in *Mtb*-HIV coinfection as well the synergy between *Mtb* and HIV during coinfection.**

1. Does HIV need to be present in the granuloma to cause functional disruption of granuloma processes? Are systemic effects of HIV enough to cause this dysfunction? Conversely, how do systemic effects induced by *Mtb* impact overall progression of both infections?
2. Does *Mtb* infection accelerate HIV infection (viral entry/pool of virus) by providing/recruiting/making available a greater diversity of cells susceptible to HIV infection? Does this greater pool of virus accelerate the depletion of cells thus breaking down the granuloma and exposing *Mtb*?
  a) On what timescale does this have the greatest impact? How fast do viral mutations begin to occur? How much faster does HIV have to replicate than normal to have an impact on the granuloma?
3. What impact does cellular recruitment have on viral load in the granuloma? If you turned recruitment down could this control viral load?
4. Is the shift of the Th phenotype of T cells to more pro-inflammatory cells induced by HIV enough to cause granuloma disruption?
5. What are the impacts of dysfunctional *Mtb-*specific CD4+ T cells in the granuloma? Does HIV-induced impairment occur at the granuloma or are they recruited already functionally impaired? How does the timing of T cell activation (through either HIV or *Mtb* induced immune activation) impact granuloma structure? Does the timing of *Mtb*-specific CD4+ T cell depletion or impairment relative to the timing or stage of *Mtb* infection affect its impact on bacterial burden?
6. What impact does macrophage phagocytosis, inability to apoptose, dendritic cell antigen processing and presentation have on pre-existing granulomas and the formation of new granulomas?
7. What are the most important time points to look at? Can this better inform when to necropsy animals?
8. What impact does the timing of infection by HIV or *Mtb* (days, weeks, months, years) have on the nature of their interaction?
9. Does *Mtb* granuloma location impact the sequence of events or influence the severity of a specific mechanism more so in one place compared to another?
10. What is the percentage of cells within the granuloma that are infected with HIV? Are there single cells coinfected with both HIV and *Mtb*, and what impact does that have on coinfected cells?
11. What type of cells are directly leading to cytokine dysregulation? Is it a lack of particular cells? What is the role of innate immunity in this cytokine dysregulation?
12. How does coinfection impact movement of cells? How does cellular movement impact granuloma structure?

## Potential impact and use of computational modeling

Computational and mathematical modeling can bolster laboratory experiments by integrating diverse types of experimental data to test and generate new hypotheses, mechanisms, dynamics, and therapeutic implementations that may be difficult and expensive to study experimentally (86). A well-validated computational model can simulate long term experimental studies in a fraction of the time and can analyze the combined impact of multiple mechanisms at once to narrow down potential experimental approaches and interventions (85). Several modeling techniques have led to insights into host-pathogen dynamics and effective treatment predictions, including multiscale models, stochastic models, game theory, continuous single- or multi-variable models, and machine learning algorithms (87). Multiscale models in particular are able to integrate information across multiple scales (molecular, cellular, tissue, organism, population) and time (seconds, minutes, hours, days, years) which enables a holistic view and approach to understanding infectious disease dynamics (87, 88). Mechanistic models can also help explore the genetic variations among animals and pathogens used. Computational modeling has been used successfully in several aspects of infectious diseases such as etiology, pathogenesis and cellular interactions [reviewed in (87)]. Computational models have been successfully applied to study separate infections with *Mtb* [reviewed in (86, 89)] or HIV [reviewed in (90)]. These established models provide a strong computational foundation and are primed to be combined to investigate the unique synergy between *Mtb* and HIV.

In the context of *Mtb*-HIV coinfection, a number of models have been developed. Kirschner 1999 developed the first mathematical model of *Mtb*-HIV coinfection dynamics using ordinary differential equations (ODEs) to focus on the effect that *Mtb* has on HIV infection with a smaller discussion on how HIV accelerates the resurgence of TB (91). Results from the model showed that in the context of coinfection, the T cell population was lower than just HIV alone, bacterial and viral loads were higher than either infection alone, and that HIV activated LTBI by causing a sharp increase in bacterial burden (91). Bauer et al., 2008 expanded on Kirschner’s 1999 model by exploring the impact of HIV on LTBI and specifically describes the time course of the adaptive immune response in coinfection. The model included more mechanistic details about immune responses such as the interactions between T cells and macrophages and the cytokine environment that they create (92). The authors concluded, in line with the experimental studies outlined above, that coinfection completely alters the cytokine environment, macrophage decline is correlated to CD4+ T cell decline and increased viral loads, and that these mechanisms result in lower recruitment to the site of infection allowing the reactivation of TB (92). Magombedze et al., 2008 developed a model (93) similar to Bauer et al., 2008 but without cytokines, and expanded it in 2010 to include ART treatment, TB treatment, and simultaneous treatment of both pathogens. They concluded, in line with current recommendations (94), that simultaneous treatment was the best option (95). Ramkissoon et al., 2012 expanded on these models to explore a variety of TB and HIV treatment strategies at various stages of HIV progression (96). Mufudza et al., 2016 incorporated HIV specific cytotoxic T cell mechanisms (not *Mtb* specific CTLs) into the model described by Kirschner 1999. Results showed that both the lytic and non-lytic factors of the HIV-specific CTLs are important in controlling HIV infection in coinfected individuals and the authors advocate for drugs and/or vaccines that enhance CTL mechanisms (97). **Table 1** highlights the important features of each model and key results obtained from the simulations. While these models offer simplified representations of the complexity of *Mtb*-HIV coinfection, they are able to recapitulate key observations from experimental studies: higher CD4+ T cell depletion than HIV infection alone, larger viral and bacterial growth than compared to singular infections, HIV reactivation of LTBI, and lower recruitment of cells to site of infection inducing reactivation of TB. The models including drugs have highlighted the risks associated with using TB and HIV drugs together in *Mtb*-HIV coinfected individuals. This highlights the need to develop safer and effective treatment strategies specifically for coinfected individuals.

**Table 1:** Computational Models of *Mtb*-HIV coinfection.

First author, year, ref #	Features	Key Results
Kirschner (1999) (91)	• CD4+ and CD8+ T cells • One macrophage population • One compartment – lymph tissue • Latent stage HIV disease • Restrict growth rate and enhance bacterial death to mimic TB drug treatment • Periodic function to observe non-compliance treatment adherence • Assume lymph and periphery are in parallel • No *Mtb*-HIV coinfected cells	• T cell populations are lower in *Mtb*-HIV coinfection than just HIV infection • Increased viral and bacterial loads during coinfection compared to single pathogen infection • Treatment that perturbs bacterial growth rate will be more effective than perturbing bacterial death rate • T cells and macrophages rebound while viral population decreases • Non-compliance can cause major complications
Bauer (2008) (92)	• 5 macrophage populations: resting, activated, *Mtb-*infected, HIV-infected, coinfected • 6 T cell populations: resting CD4+, Th1, HIV-1 infected CD4+, resting CD8+, HIV-1 specific cytotoxic, and *Mtb* specific cytotoxic • Intra- and extracellular *Mtb* • 5 cytokines: TNFα, IFNγ, IL-10, IL-12, IL-4 • One compartment – lung • Fix TB related parameters to result in LTBI while varying HIV related parameters • Lymph node dynamics captured as first step	• Lower CD4 and CD8 counts in coinfection than single pathogen infection • Rapid reactivation of LTBI to active disease • Delay in peak viral load followed by lower set point in coinfection than just HIV infection o Likely due to no fluctuations in HIV-specific CTL population in HIV infection alone
Magombedze (2008) (93)	• 3 macrophage populations: resting, *Mtb* infected, HIV-infected • 2 CD4+ T cell populations: HIV-infected and HIV-uninfected • 2 CTL populations: *Mtb*-specific and HIV-specific • Model TB and HIV simultaneously • Latent TB infection then HIV infection • HIV infection then TB infection • One compartment – lung • No *Mtb*-HIV coinfected cells	• Increased rate of immune cell depletion in coinfection • Coinfection accelerates first pathogen to cause infection (true for both TB infection first and HIV infection first) • Highest depletion of resting macrophages occurs from coinfection that starts from latent *Mtb* infection • More rapid decline of resting macrophages occurs in coinfection that starts from HIV infection • Rapid bacterial growth in coinfection starting from *Mtb* latency • *Mtb* infected macrophages rise slowly in *Mtb* infection after HIV infection but proceed to active TB unlike in *Mtb* only infection • Higher viral infection of macrophages occurs during coinfection than HIV infection alone
Magombedze (2010) (95)	• 5 macrophage populations: resting, *Mtb* infected, exposed to HIV and infected by HIV • 3 CD4+ T cell populations: uninfected/susceptible, infected/exposed, and productively infected • 2 CTL populations: *Mtb*-specific and HIV-specific • Slight modifications to some equations from Magombedze et al., 2008 o Inclusion of HIV transcription mechanisms in HIV infection equations and coinfection equations o Simulation of HIV and TB drugs (3 of each) • No *Mtb*-HIV coinfected cells	• Simultaneous treatment with TB and HIV drugs reduces performance of HIV drugs • Best treatment of TB in coinfection is simultaneous TB and HIV drugs • Best treatment of HIV in coinfection is just HIV drugs without TB drugs • Improving performance of RTIs in CD4+ T cells shows decrease in infected CD4+ T cells but increase in HIV-infected macrophages • As HIV drug performs better in CD4+ T cells, HIV might selectively multiply in macrophages
Ramkissoon (2012) (96)	• 5 macrophage populations: resting, activated, *Mtb*-infected in eclipse stage, *Mtb*-infected in productive stage, HIV-infected • 3 CD4+ T cells: susceptible, HIV-infected in eclipse stage, productively infected • 2 CD8+ (CTLs) populations: HIV-specific and *Mtb*-specific • No *Mtb*-HIV coinfected cells • Simulates HIV disease progression – early, late, AIDS • Inhibition of bacterial growth or bacterial death to simulate TB drugs (2 drugs) • ART is included by term that reduces HIV infection with modification for TB-HIV drug interaction (2 drugs)	• Treating HIV first is not a generally good strategy • Potential risks of drug-drug interactions negate early ART benefits leading to increased viral load and suppressed CD4+ T cell count • In early and late HIV disease, administering TB treatment first then ART 6 months after TB treatment has low risk for AIDS death, TB death, drug overlap, and TB-IRIS
Mufudza (2016) (97)	• Addition of HIV-specific CTL to Kirschner 1999	• Non-lytic mechanisms are more effective in controlling infection when the rate of CTL mediated killings is low relative to the rate of cell-mediated killings • Lytic mechanisms are more important when the rate of virus induced CD4+ T cell deaths is high relative to the rate of CTL mediated killing regardless of the number of CTLs • Lytic arm is more important to control infection • Non-lytic arm is more important to control virus replication • Presence of CTLs helps to reduce viral loads and maintain CD4+ T cell numbers

However, as we discussed above, to fully take advantage of computational models to answer key questions about *Mtb*-HIV synergy, one must account for the structure, function and organization of granulomas. Currently all of the models for *Mtb*-HIV coinfection are non-spatial and represent the lung or lymph tissue compartment as a whole which does not allow them to describe spatial granuloma dynamics. ODE models assume the populations are homogenous and uniformly distributed (98). Therefore, they can describe average population behaviors within coinfection, but they are not always suitable for systems where spatial aspects are important for dynamics as in the case of the *Mtb* granuloma. An alternative modeling method that provides this spatial resolution are agent-based models (ABMs). ABMs are stochastic models well-suited for modeling granuloma formation and function as they can incorporate spatial dynamics that ODEs cannot. ABMs describe emergent tissue-level behavior resulting from cellular-level rules and interactions. Individual agents dynamically and independently respond to environmental cues (98). ABMs of immune responses and inflammation have been successfully applied to evaluate sepsis, asthma, hepatic infection, pulmonary fibrosis, wound healing/soft tissue injury, *in vivo* granuloma formation in TB infection, cancer/oncogenesis, angiogenesis, and gastrointestinal infection/microbiome [reviewed in (99)]. Several models explore the temporal and spatial aspects of TB granulomas (17, 79, 100, 101). Such spatial ABMs are well-positioned to quantify how the mechanisms presented in this review interact within the granuloma and tissue microenvironments and their relative contributions to granuloma level outcomes such as size, bacterial and viral load, cytokine profile, as well as frequency, location and functional status of immune cells. Computational models enable knockout simulations by removing a mechanism, cell type, or parameter to quantify the impact of depletion or deletion on granuloma level outcomes. Furthermore, global sensitivity analyses of computational models allow for the quantification of individual mechanism impacts in the context of multiple interacting mechanisms. **Box 2** highlights several examples of how the outstanding questions posed in **Box 1** can be answered by leveraging the features of computational models.

It is important to note that mechanistic computational models are limited to what mechanisms or biological features are already known about the system. Computational models are a simplification of the real system limited to what is currently known about the system and also by the question being answered, and therefore cannot capture all of the biological complexity. Computational models can also be difficult to parametrize and therefore it is critical to quantify parameter uncertainty to understand how parameter uncertainty impacts the results. Performing global uncertainty and sensitivity analyses can help clarify the limitations of the model and inform interpretation of simulation results.

**Box 2: Attributes of computational models that can help address outstanding questions posed in Box 1.**

**Table:** 

**Model Feature**	**Questions in Box 1**
Control what infection is first and the condition of the single infection progression (characterized by bacterial load, viral load, cell numbers, granuloma size or quantity, time, etc.) before adding the second infection	2a, 3, 5, 6, 7, 8
Virtual knockouts of various cell types to determine the influence the absence of specific cells have on the granuloma structure	3, 4, 5, 10, 11
Virtual knockouts of mechanisms through parameter tuning. Rates of secretion, phagocytosis, bacterial killing, and viral replication, to name a few, could all be varied to understand their impact of the maintenance of the granuloma	2, 4, 5, 6, 10, 11, 12
Uncertainty/sensitivity analysis to determine which parameters have the most influence on model outputs.	3, 4, 5, 6, 7, 8
Predict how mechanisms or outputs in one location or timepoint influences a near or far location	1, 6, 7, 8, 9

## Conclusions

It is widely accepted that *Mtb* and HIV synergistically disrupt the immune system and exacerbate the burden of both diseases. Specific interactions between *Mtb* and HIV include: disruption of the pro- and anti-inflammatory cytokine balance, chronic immune activation, impairment of CD4+ T cell and *Mtb*-specific T cell functionality, *Mtb*-induced increase in HIV viral load and viral diversity, and HIV impairment of innate immune cells. A key result of these interactions during *Mtb*-HIV coinfection is the disruption of the structure and function of granulomas. There exists a need for an inexpensive and versatile method for exploring and quantifying *granuloma-specific* outcomes resulting from *multiple interconnected* mechanisms of interaction between *Mtb* and HIV. Computational modeling of *Mtb* granulomas in *Mtb*-HIV coinfection in conjunction with *in vitro* and *in vivo* studies can provide such methods. While these mechanisms have been demonstrated independently of eachother, computational models can combine multiple mechanisms to quantify the effect of each mechanism, through: parameter tuning, uncertainty and sensitivity analysis, and virtual knockouts. Building reliable and informative models will require a collaborative, iterative communication and data exchange between microbiologists, immunologists and computational biologists. Such interdisciplinary approaches will accelerate and advance a quantitative understanding of the complex, spatial and multi-scale synergies between *Mtb* and HIV within the granuloma.

## Author contributions

All authors contributed to the conceptualization of the work. AH produced the original draft of the manuscript, and all authors reviewed and edited the manuscript. AH produced the visualization. All authors contributed to the article and approved the submitted version.

## Funding

This work was funded by a PhRMA Foundation Research Starter Grant to EP and LS and NIH R21 AI145539 to LS.

## Conflict of interest

The authors declare that the research was conducted in the absence of any commercial or financial relationships that could be construed as a potential conflict of interest.

## Publisher’s note

All claims expressed in this article are solely those of the authors and do not necessarily represent those of their affiliated organizations, or those of the publisher, the editors and the reviewers. Any product that may be evaluated in this article, or claim that may be made by its manufacturer, is not guaranteed or endorsed by the publisher.
